# Supplementary figures and images for: Genome-wide association studies reveal the role of polymorphisms affecting factor H binding protein expression in host invasion by Neisseria meningitidis
Source: PLoS Pathog. 2021 Oct 18;17(10):e1009992. doi: 10.1371/journal.ppat.1009992 (PMC8553145; doi:10.1371/journal.ppat.1009992)

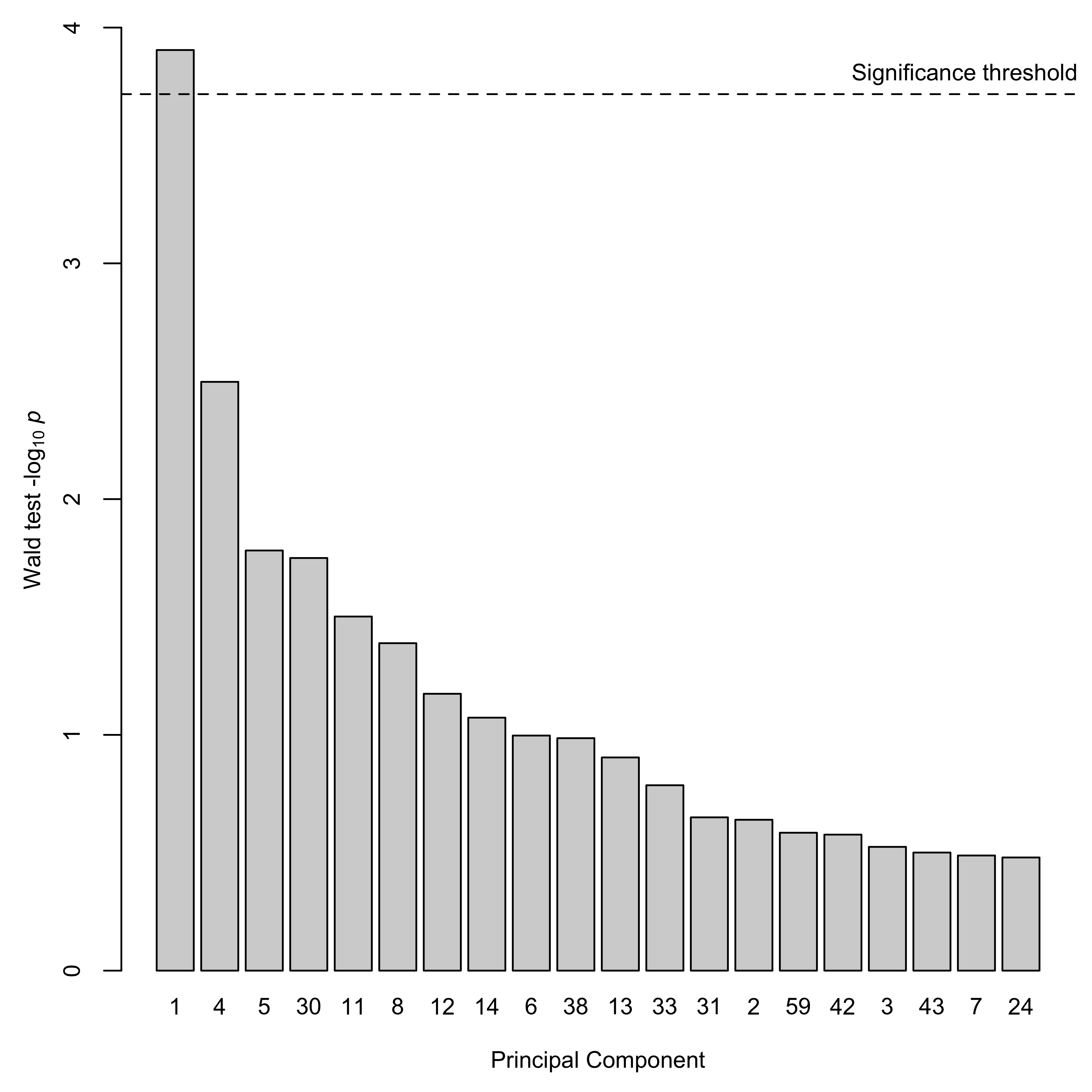

Supplement: S1 Fig — A Bonferroni correction was applied to the significance threshold for the number of non-redundant PCs. (PNG) [file ppat.1009992.s001.png]

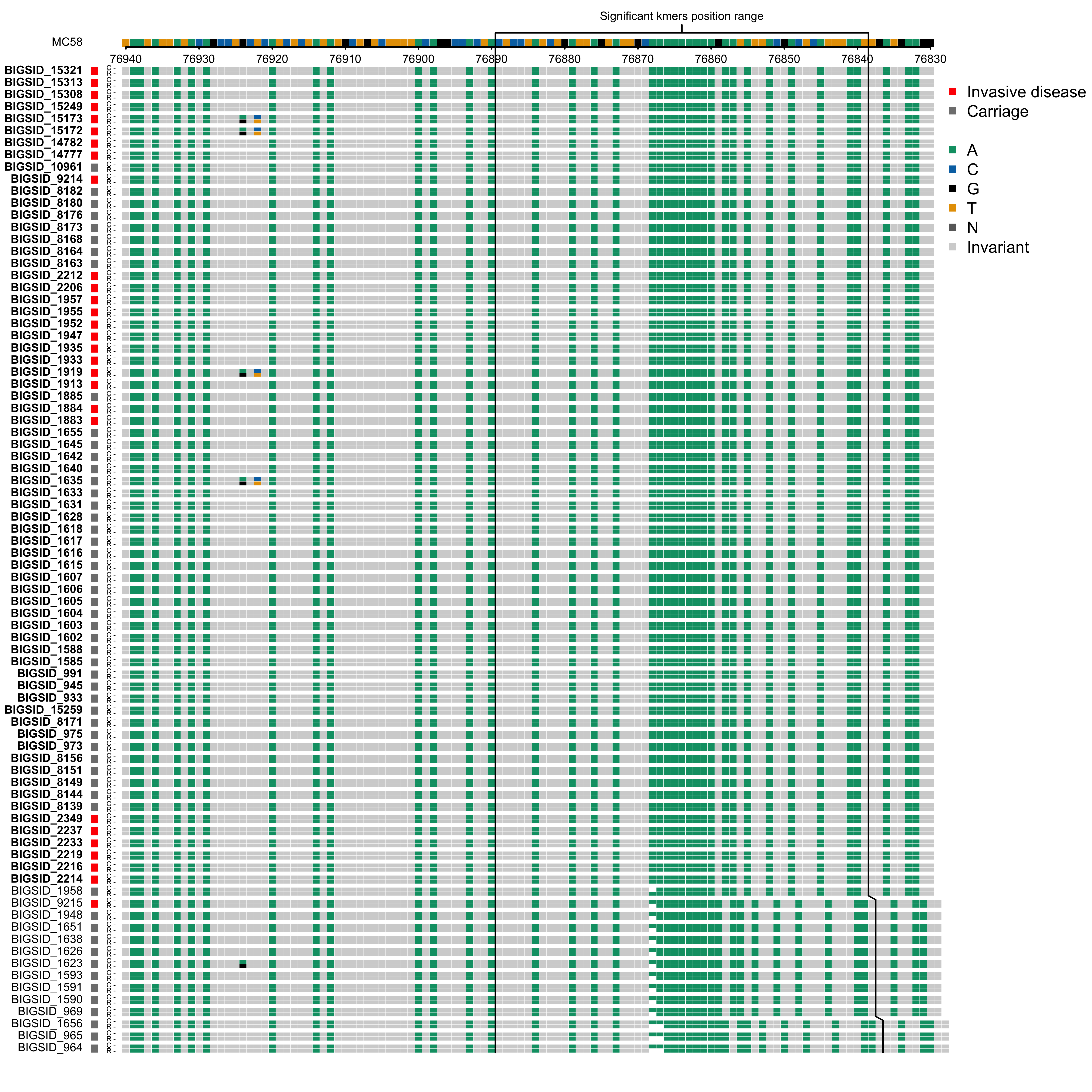

Supplement: S2 Fig — Each row shows an alignment of a contig (C) with the reference (R) from BLAST (Camacho et al. 2009). On the left, red indicates that the isolate was sampled from a patient with invasive disease, grey from a carrier. In the alignments, grey indicates identity between the contig and the reference. Polymorphisms are coloured by the alleles (A = green; C = blue; G = black; T = Orange) plus all invariant positions with the A allele are coloured green. Insertions and deletions are shown in white. The top line shows the bases of the MC58 csb gene in the region. Vertical lines show the region where the 21 significant csb kmers mapped. Sample names are shown as BIGS IDs from pubMLST and sample names shown in bold contained the 21 significant kmers. (PNG) [file ppat.1009992.s002.png]

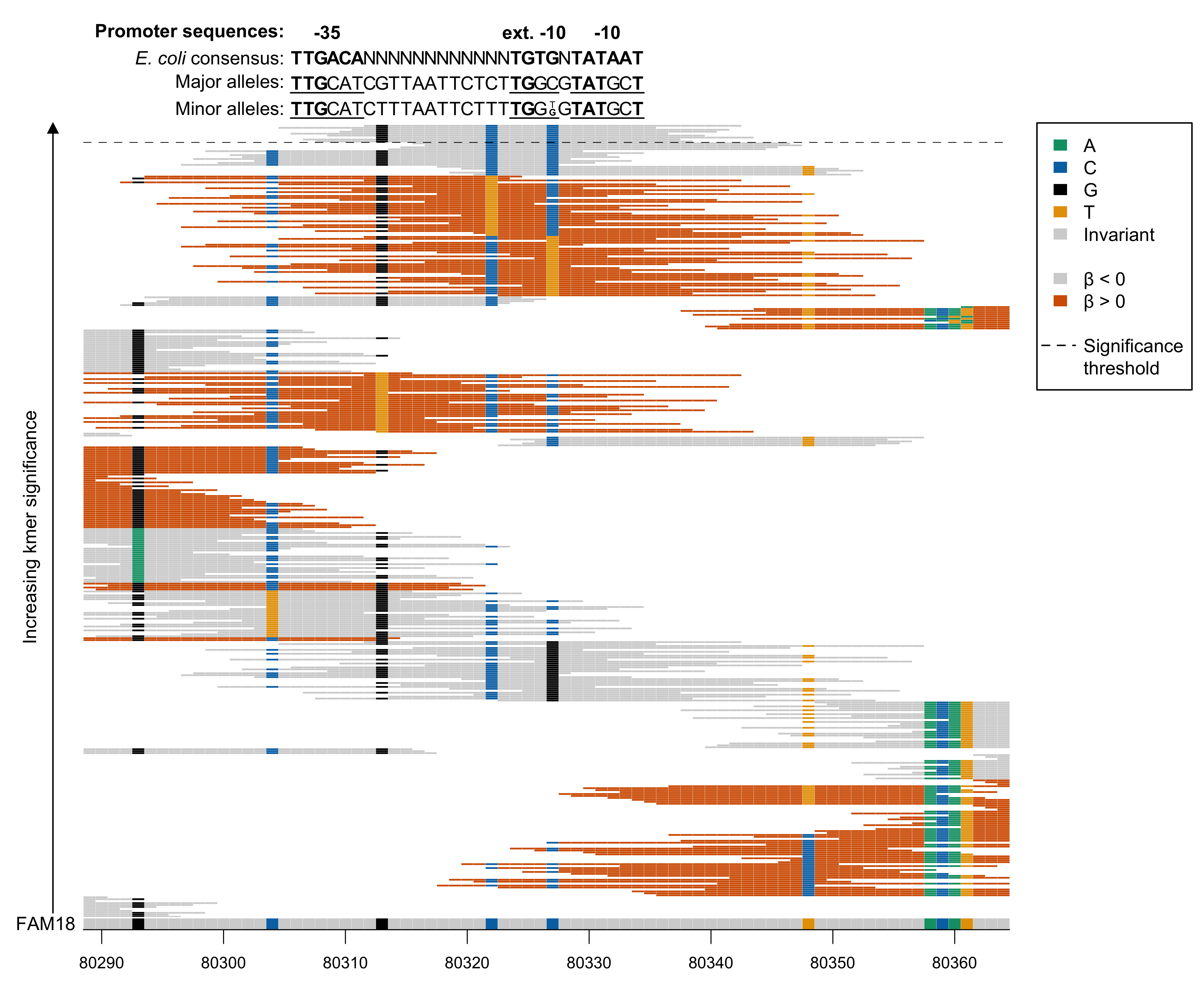

Supplement: S3 Fig — The reference genome FAM18 is shown at the bottom of the figure, grey for invariant sites and coloured at variant site positions. The kmers which map to the region shown are then plotted from least significant at the bottom to most significant at the top. The black dashed line indicates the Bonferroni-corrected significance threshold–all kmers above the line are significantly associated with the phenotype. The background colour of the kmers represents the direction of the association, grey when β < 0 (carriage-associated) and dark orange when β > 0 (disease-associated). Kmers are coloured by their allele at all variant positions (A = green; C = blue; G = black; T = Orange). The E. coli consensus for the -10 and -35 promoter regions are shown above the kmers aligned with the major and minor alleles in the discovery sample collection at these positions. Matches to the consensus are shown in bold. (PNG) [file ppat.1009992.s003.png]

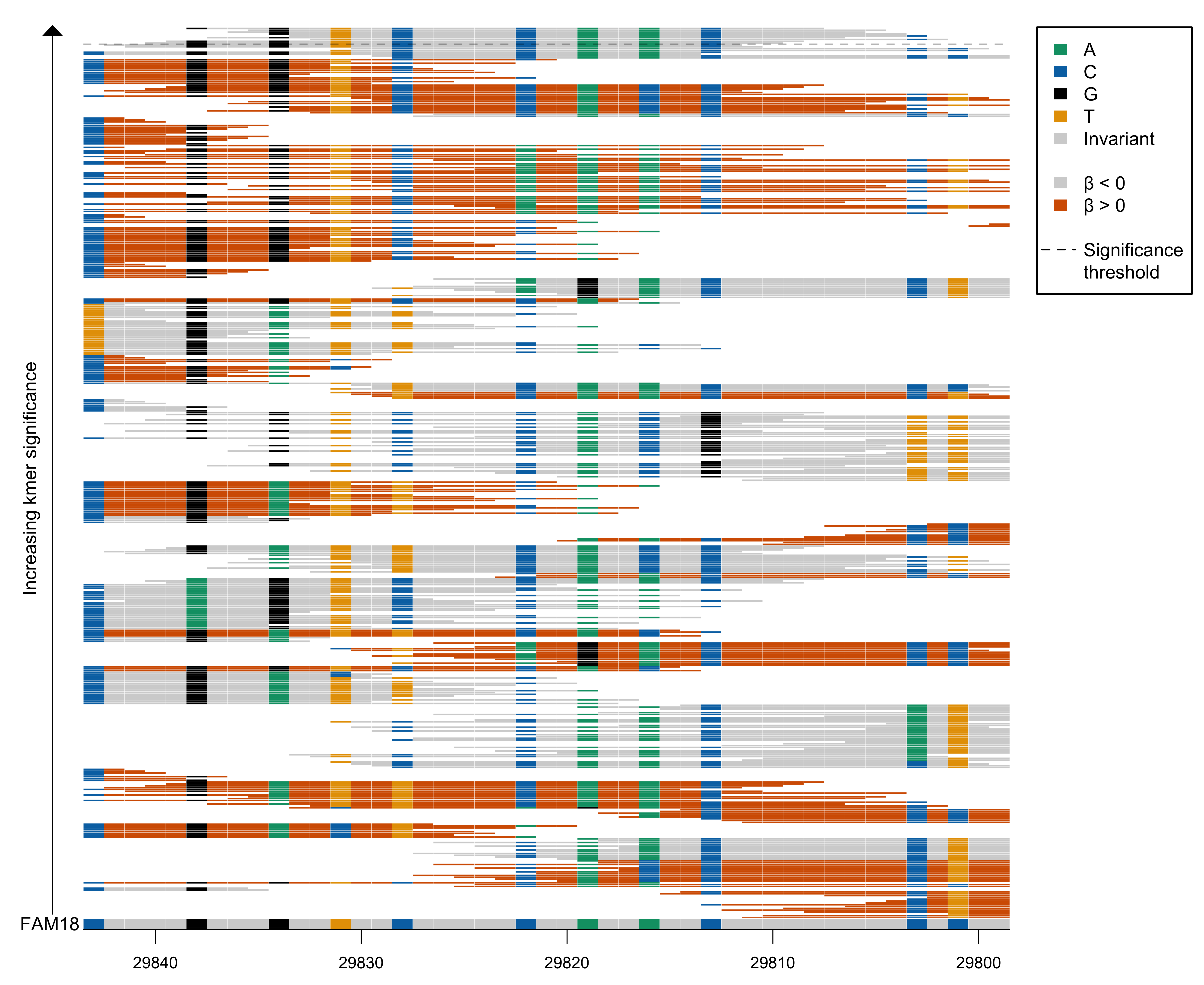

Supplement: S4 Fig — The reference genome FAM18 is shown at the bottom of the figure, grey for invariant sites and coloured at variant site positions. The kmers which map to the region shown are then plotted from least significant at the bottom to most significant at the top. The black dashed line indicates the Bonferroni-corrected significance threshold–all kmers above the line are significantly associated with the phenotype. The background colour of the kmers represents the direction of the association, grey when β < 0 (carriage-associated) and dark orange when β > 0 (disease-associated). Kmers are coloured by their allele at all variant positions (A = green; C = blue; G = black; T = Orange). (PNG) [file ppat.1009992.s004.png]

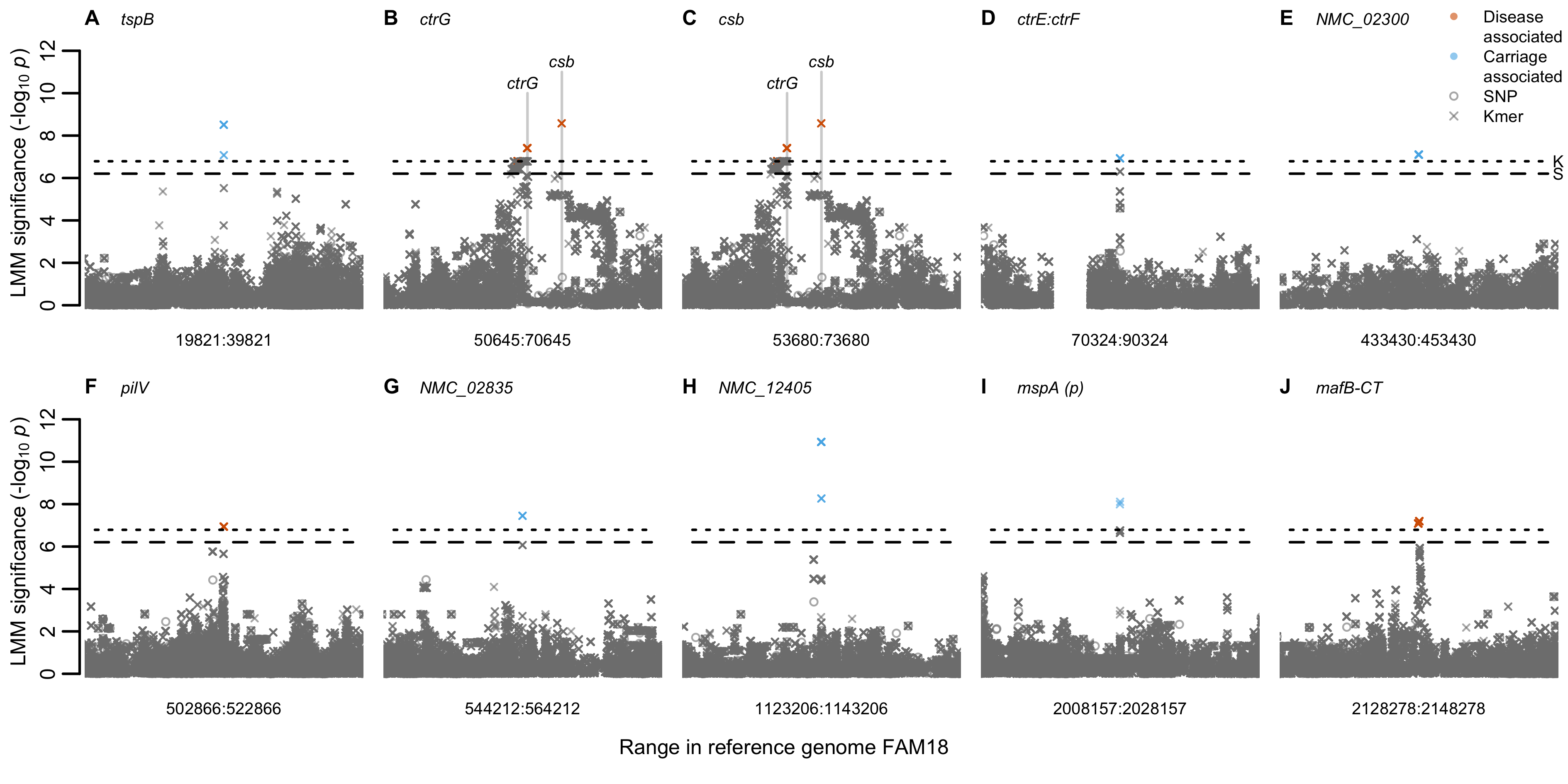

Supplement: S5 Fig — Each plot shows the midpoint of the kmer association within the gene/intergenic region +/- 10kb. Open circles represent a SNP aligned to the reference genome FAM18, a cross represents the left-most mapping position of a kmer in the reference genome FAM18 based on mapping and BLAST alignments. Significant kmers are coloured by the LMM estimated direction of effect. Bonferroni-corrected significance thresholds are shown by black dashed (SNPs) and dotted (kmers) lines. Gene names separated by colons indicate intergenic regions. FAM18 reference genome gene name prefixes have been shortened from NMC_RS to NMC_. (PNG) [file ppat.1009992.s005.png]

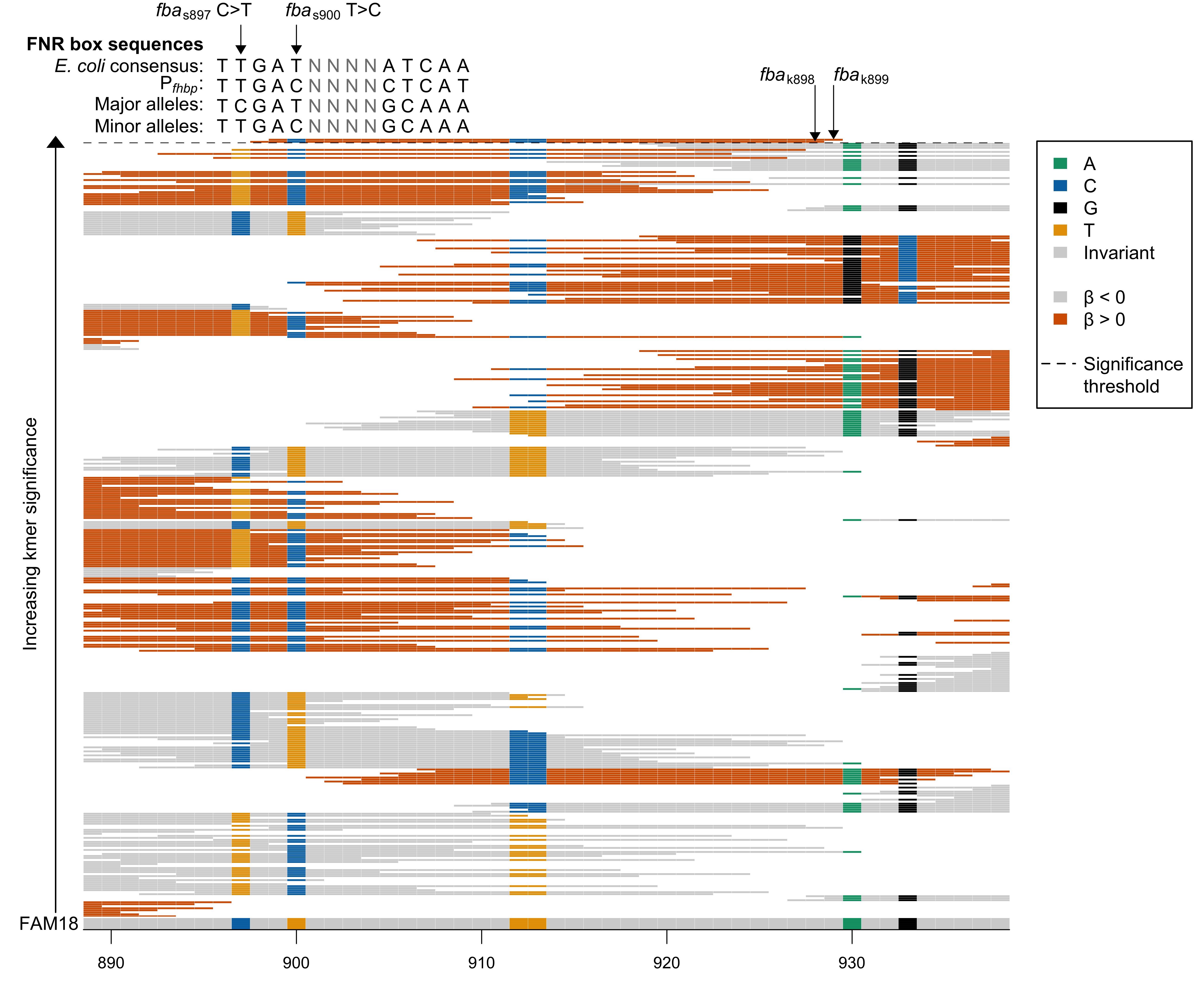

Supplement: S6 Fig — The reference genome FAM18 is shown at the bottom of the figure, grey for invariant sites and coloured at variant site positions. The kmers which map to the region shown are then plotted from least significant at the bottom to most significant at the top. The black dashed line indicates the Bonferroni-corrected significance threshold–all kmers above the line are significantly associated with the phenotype. The background colour of the kmers represents the direction of the association, grey when β < 0 (carriage-associated) and dark orange when β > 0 (disease-associated). Kmers are coloured by their allele at all variant positions (A = green; C = blue; G = black; T = Orange). The E. coli consensus for the FNR box DNA binding site and the fHbp promoter FNR binding site are shown above the kmers aligned with the major and minor alleles in the discovery sample collection at these positions. (PNG) [file ppat.1009992.s006.png]

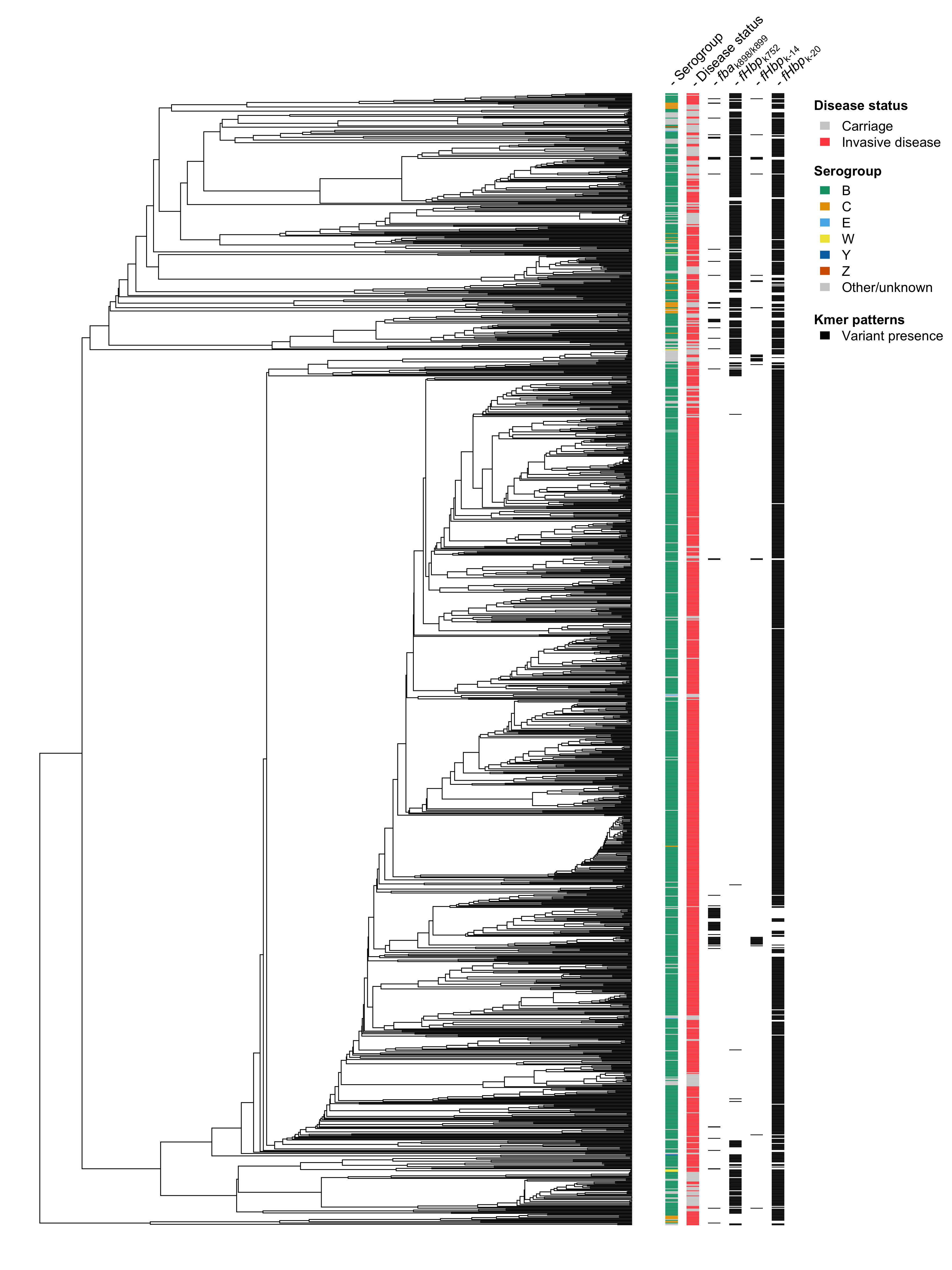

Supplement: S7 Fig — The UPGMA tree, used solely for visualisation, was estimated using a distance matrix calculated from the kmer presence/absence matrix. The most common serogroups are shown on the outer ring. Disease status is shown on the next ring, invasive disease (red, n = 1,046) or carriage (grey, n = 249). Presence of the two significant kmers in fba in the discovery sample collection are shown in black in the inner ring. (PNG) [file ppat.1009992.s007.png]

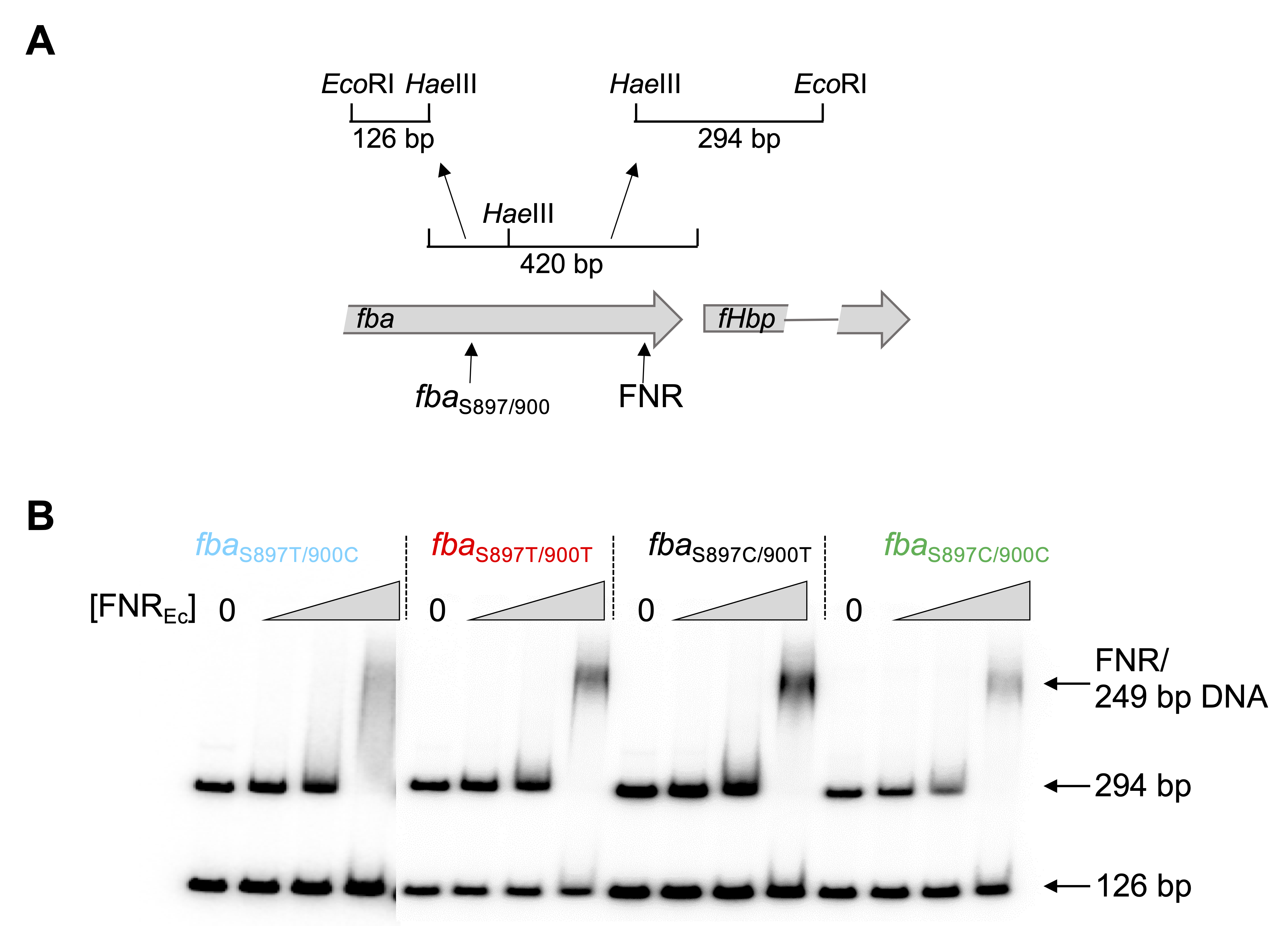

Supplement: S8 Fig — (A) The 126 and 249 bp fragments used for EMSA. (B) Sequences upstream of fHbp were amplified and digested with HaeIII, end labelled with [γ-32P]-ATP, then incubated in increasing concentrations of FNR (0, 0.75, 1.5, and 3 μM). (PNG) [file ppat.1009992.s008.png]

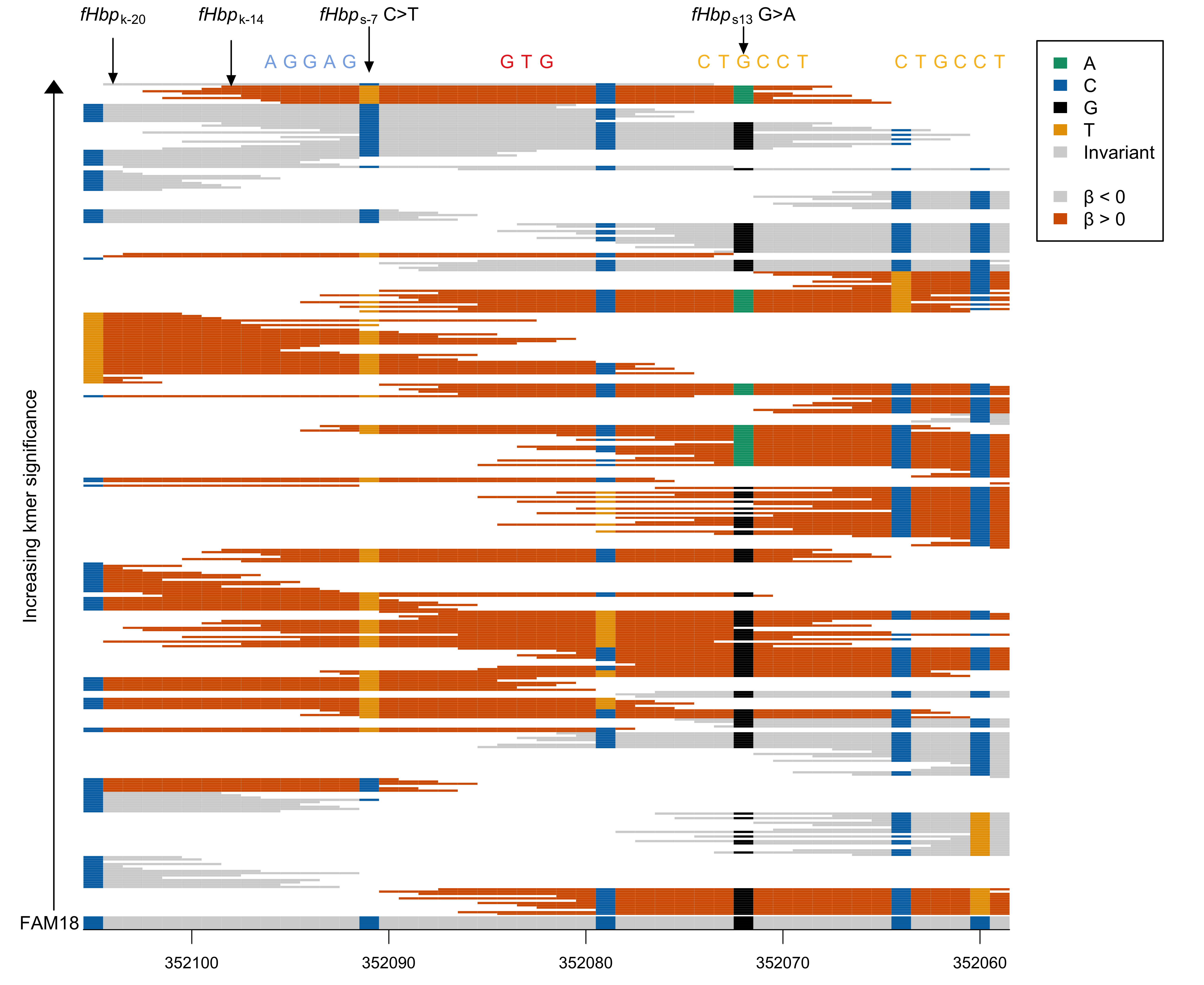

Supplement: S9 Fig — The reference genome FAM18 is shown at the bottom of the figure, grey for invariant sites and coloured at variant site positions. The kmers above 1% minor allele frequency that map or align to the region shown are plotted from least significant at the bottom to most significant at the top. The background colour of the kmers represents the direction of the association, grey when β < 0 (carriage-associated) and dark orange when β > 0 (disease-associated). Kmers are coloured by their allele at all variant positions (A = green; C = blue; G = black; T = Orange). The fHbp start codon is shown aligned above the kmers in red, the ribosome binding site (RBS) in blue and two putative anti-RBSs in orange. The most significant kmers plus the SNPs tested experimentally are labelled. (PNG) [file ppat.1009992.s009.png]

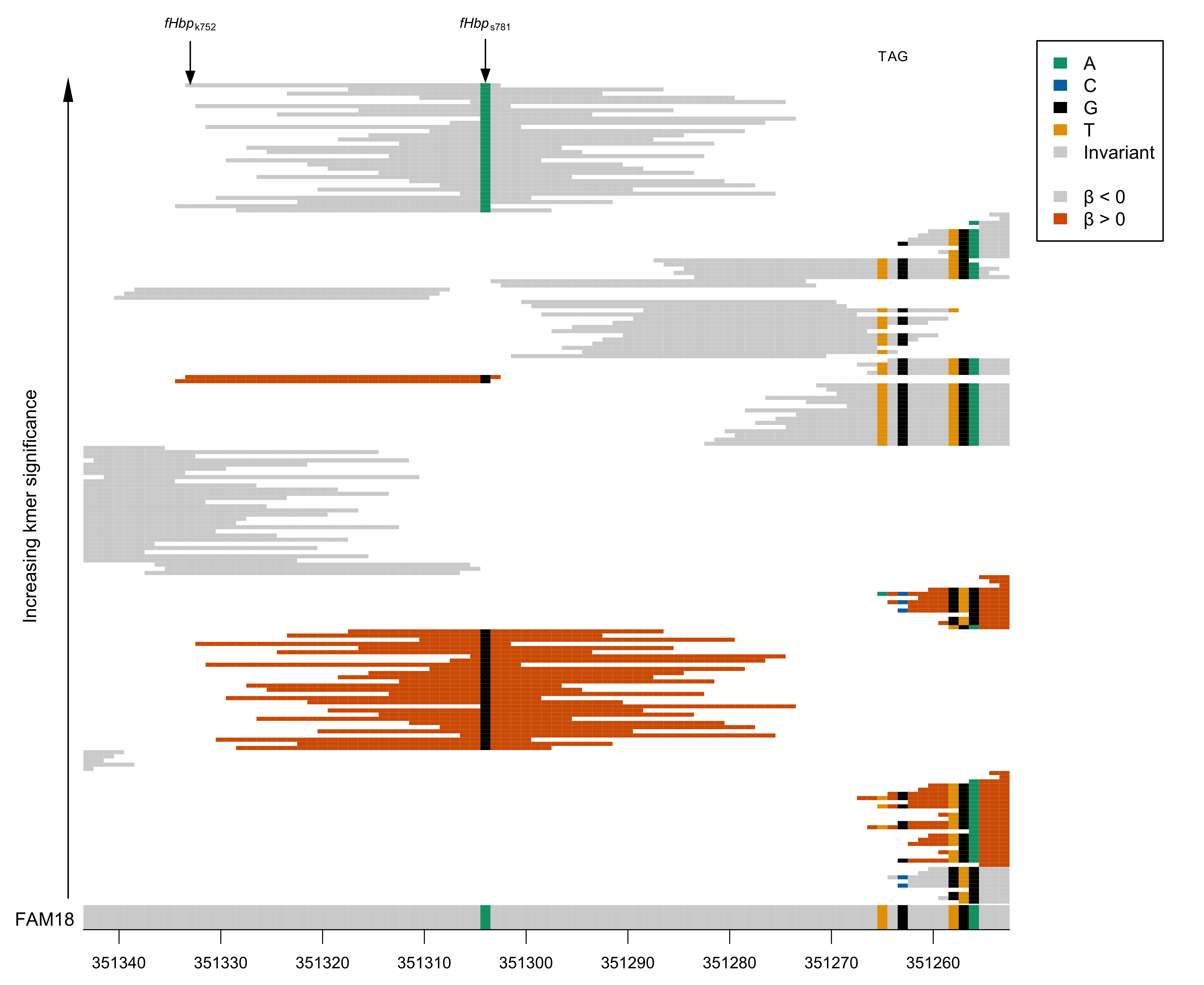

Supplement: S10 Fig — The reference genome FAM18 is shown at the bottom of the figure, grey for invariant sites and coloured at variant site positions. The kmers which map to the region shown are then plotted from least significant at the bottom to most significant at the top. The background colour of the kmers represents the direction of the association, grey when β < 0 (carriage-associated) and dark orange when β > 0 (disease-associated). Kmers are coloured by their allele at all variant positions (A = green; C = blue; G = black; T = Orange). The fHbp stop codon is annotated above the aligned kmers. The most significant kmer plus the SNP tested experimentally are labelled. (PNG) [file ppat.1009992.s010.png]

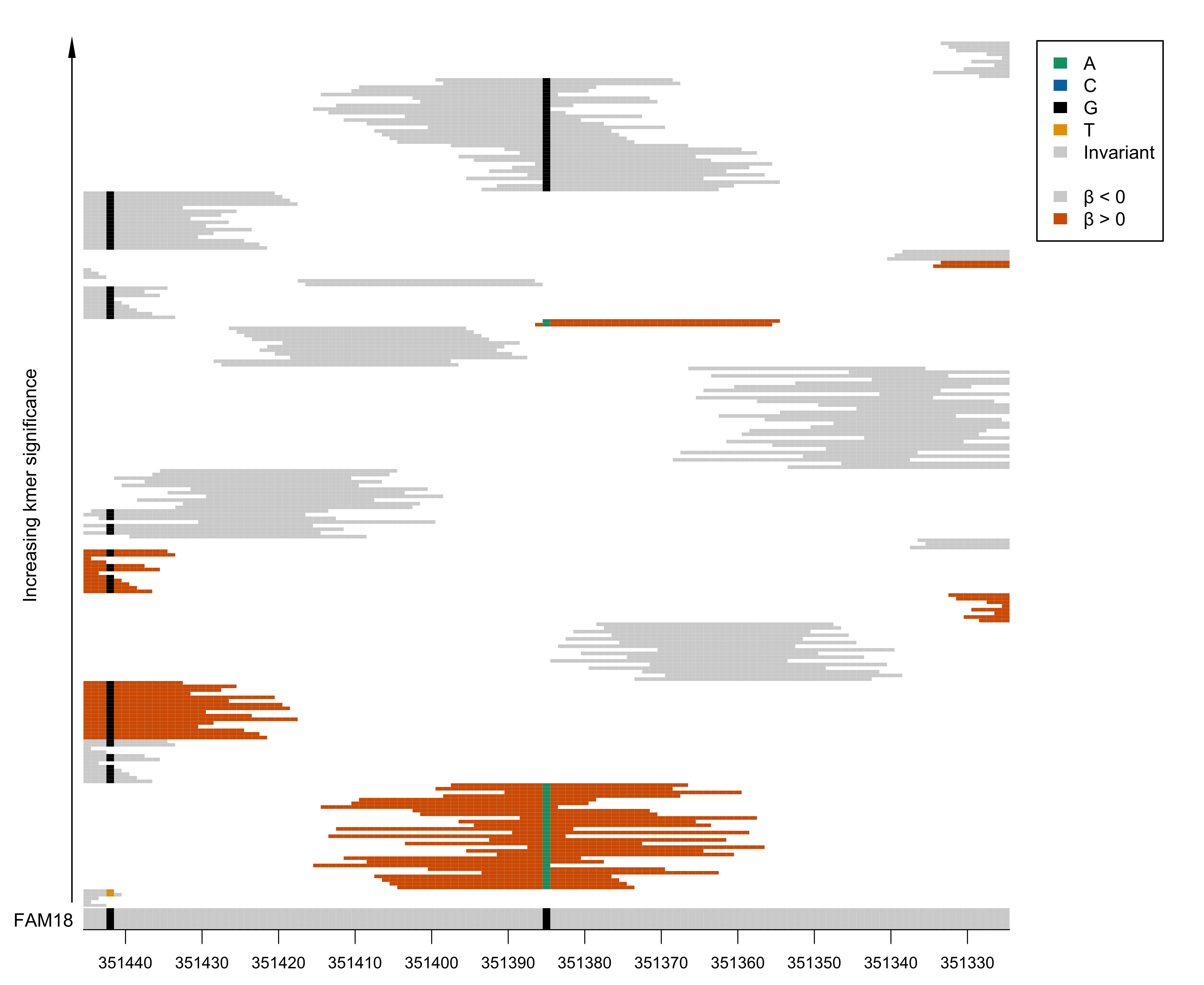

Supplement: S11 Fig — The reference genome FAM18 is shown at the bottom of the figure, grey for invariant sites and coloured at variant site positions. The kmers which map to the region shown are then plotted from least significant at the bottom to most significant at the top. The background colour of the kmers represents the direction of the association, grey when β < 0 (carriage-associated) and dark orange when β > 0 (disease-associated). Kmers are coloured by their allele at all variant positions (A = green; C = blue; G = black; T = Orange). The fHbp stop codon is annotated above the aligned kmers in red. (PNG) [file ppat.1009992.s011.png]

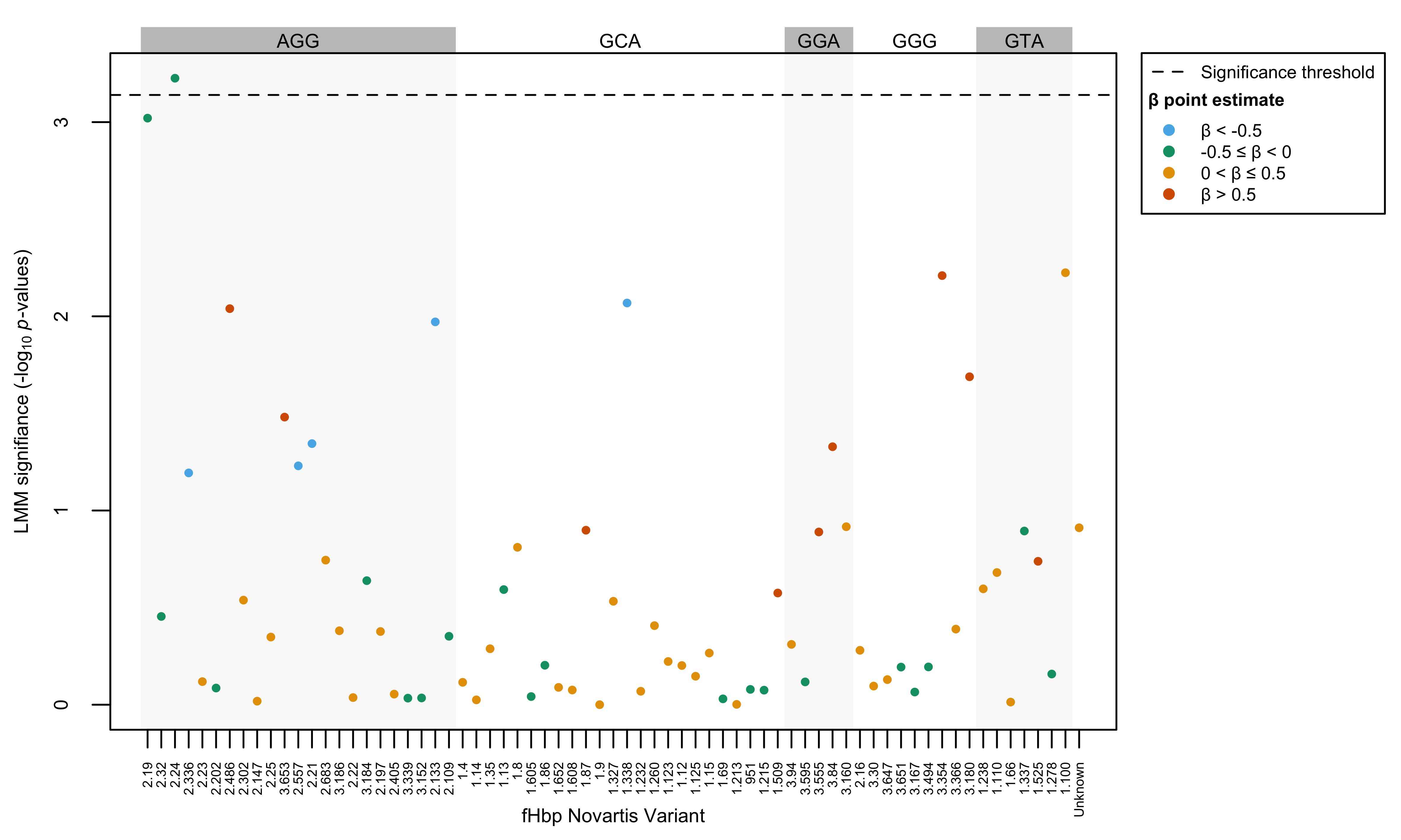

Supplement: S12 Fig — Correcting the significance threshold for the number of variants, variant 2.24 was significantly associated with disease status. The codon each variant contains at codon 261 (relative to the FAM18 reference fHbp) is shown at the top. Colour represents the β point estimate, the direction of the effect of the association by the LMM. (PNG) [file ppat.1009992.s012.png]

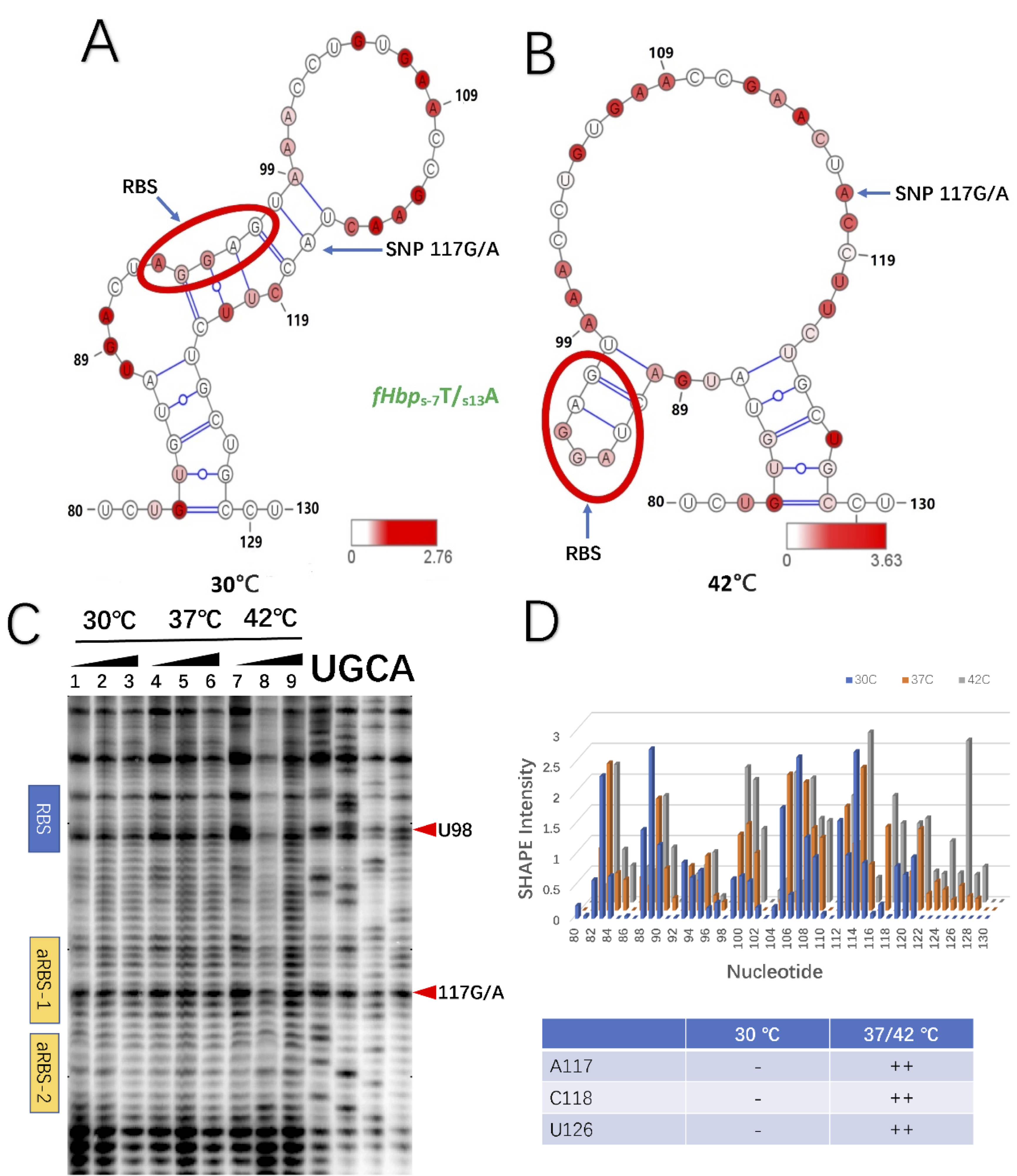

Supplement: S13 Fig — SHAPE reactivity data are mapped on the RNA structure and colour coded by intensity as shown on the bars; the RBS is circled in red. (C) NMIA modifications for reactions conducted at 30°C (Lane1- 3); 37°C (lane 4–6) and 42°C (lane 7–9), with a gradient of 0-13mM NMIA, analysed by denaturing polyacrylamide gel electrophoresis. (D) SHAPE reactivity profile at different temperatures; nucleotides with temperature dependent changes in reactivity are listed in the table as strong (++), medium (+) and weak (-). (PNG) [file ppat.1009992.s013.png]

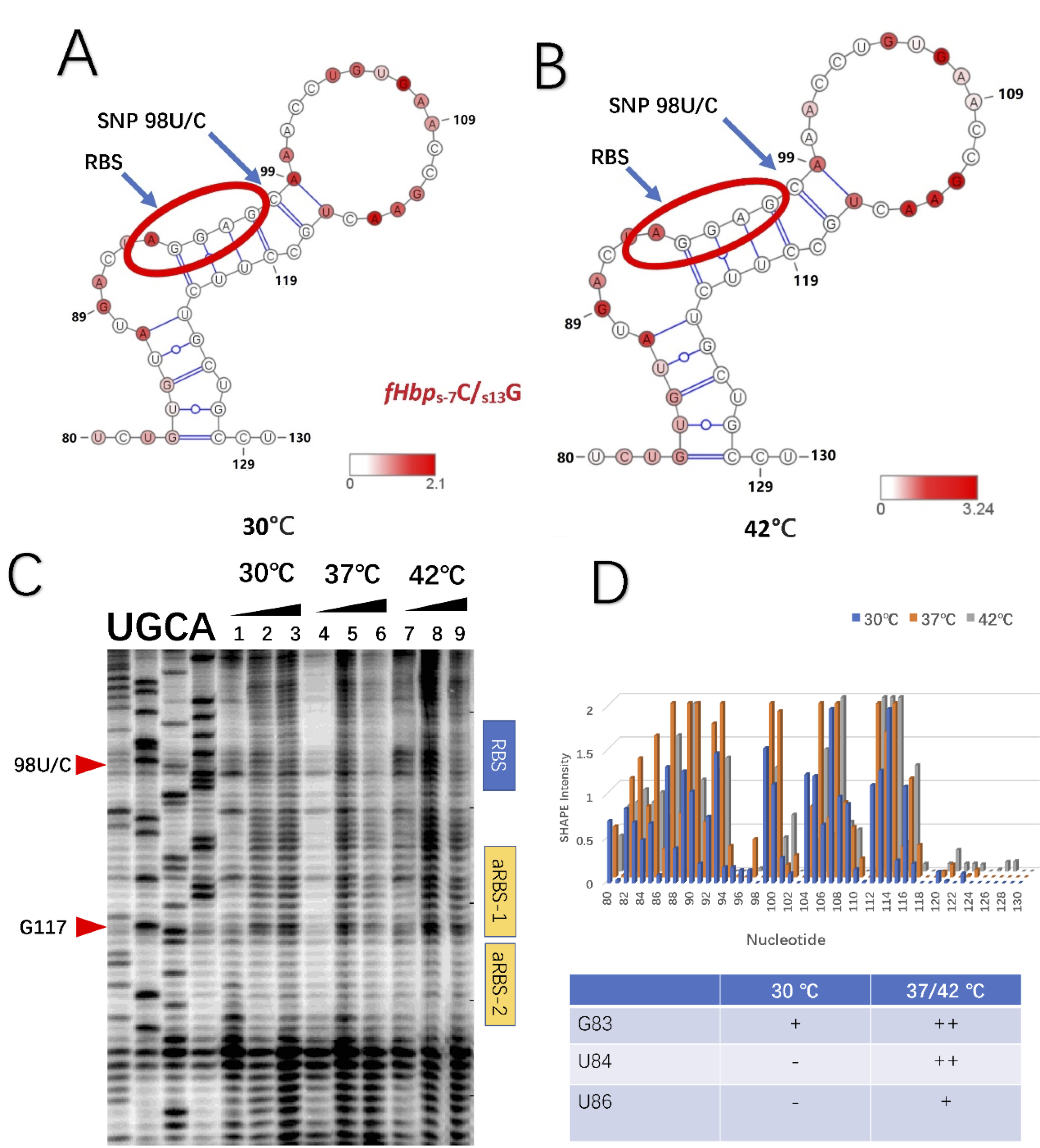

Supplement: S14 Fig — Secondary structure of the fHbps-7C/s13G RNA calculated using RNA structure based on SHAPE reactivity data at (A) 30°C and (B) 42°C; SHAPE reactivity data are mapped on the RNA structure and colour coded by intensity as shown on the bars; the RBS is circled in red. (C) NMIA modification for reactions conducted at 30°C (Lane 1–3); 37°C (lane 4–6) and 42°C (lane 7–9), with a gradient of 0-13mM NMIA, analysed by denaturing polyacrylamide gel electrophoresis. (D) SHAPE reactivity profile at the different temperatures; nucleotides with temperature dependent changes in reactivity are listed in the table as strong (++), medium (+) and weak (-). (PNG) [file ppat.1009992.s014.png]

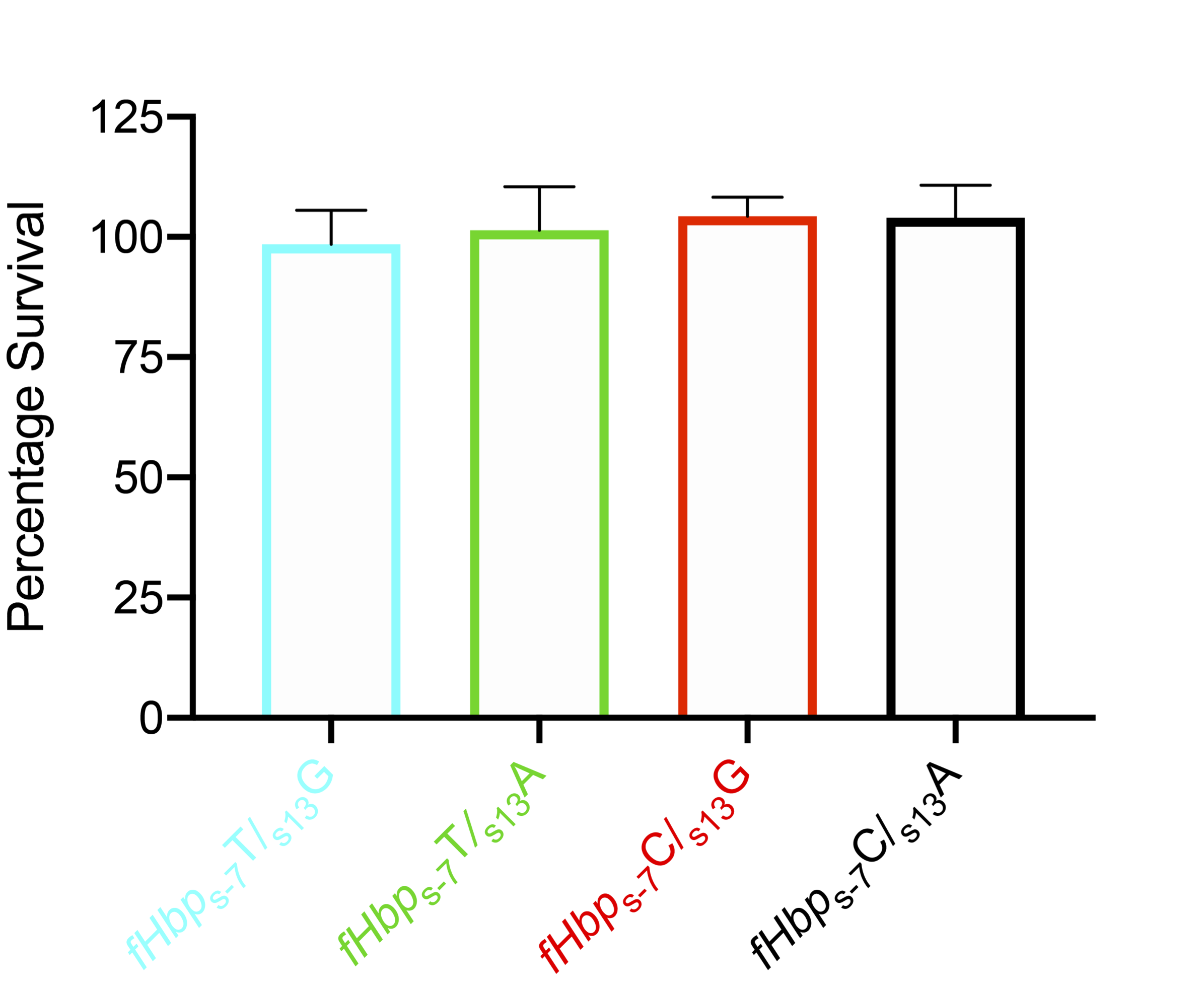

Supplement: S15 Fig — SNPs; fHbps-7T/fHbps13G (blue), fHbps-7T/fHbps13A (green), fHbps-7C/fHbps13G (red) and fHbps-7C/fHbps13A (black) demonstrated no statistical difference in bacterial survival. Error bars show SD (n = 3) with statistical analysis performed in Prism v10 using One-way ANOVA. (PNG) [file ppat.1009992.s015.png]
