# Supplementary material for: Genome-wide association studies reveal the role of polymorphisms affecting factor H binding protein expression in host invasion by Neisseria meningitidis
Source: PLoS Pathog. 2021 Oct 18;17(10):e1009992. doi: 10.1371/journal.ppat.1009992 (PMC8553145; doi:10.1371/journal.ppat.1009992)
Supplement: S1 Table — (PDF) [file ppat.1009992.s016.pdf]

S1 Table Strains used to test *fba* and *fHbp* SNPs

Species/strain	Genotype/Description	Source
E. coli		
Dh5α	F– *endA1 glnV44 thi-1 recA1 relA1 gyrA96 deoR nupG purB20* φ80d*lacZ*ΔM15 Δ(*lacZYA-argF*)U169, hsdR17($r_{K}-m_{K+}$), λ–	Invitrogen
N. meningitidis		
0011/93	Serogroup B, ST-41/44, IMD 1993	
OX99.32412	Serogroup C, ST-41/44, Carriage, 1999	
fHbp$_{S-7}$T/$_{S13}$G	0011/93 with *fHbp*$_{S-7}$T/$_{S13}$G, ery^{R} downstream of *fHbp*	This study
fHbp$_{S-7}$T/$_{S13}$A	0011/93 with *fHbp*$_{S-7}$T/$_{S13}$A, ery^{R} downstream of *fHbp*	This study
fHbp$_{S-7}$C/$_{S13}$G	0011/93 with *fHbp*$_{S-7}$C/$_{S13}$G, ery^{R} downstream of *fHbp*	This study
fHbp$_{S-7}$C/$_{S13}$A	0011/93 with *fHbp*$_{S-7}$C/$_{S13}$A, ery^{R} downstream of *fHbp*	This study
