# Supplementary material for: Genome-wide association studies reveal the role of polymorphisms affecting factor H binding protein expression in host invasion by Neisseria meningitidis
Source: PLoS Pathog. 2021 Oct 18;17(10):e1009992. doi: 10.1371/journal.ppat.1009992 (PMC8553145; doi:10.1371/journal.ppat.1009992)
Supplement: S2 Table — (PDF) [file ppat.1009992.s017.pdf]

S2 Table: Plasmids

Plasmid	*E. coli*	Description	Source
pET28a	DH5α	-	Novagen
pET21b	DH5α	-	Novagen
pET24-HIS-TEV	DH5α	TEV protease cleavage site tagged with HIS	This study
pET28a-His-MBP-TEV	DH5α	For expression of fHbps fused to MBP fusion	This study
pET21b_V2.24_A	B834	vector for IPTG inducible expression of fHbp $V2.24^{261R}$	This study
pET21b_V2.24_G	B834	vector for IPTG inducible expression of fHbp $V2.24^{261G}$	This study
pET21b_$V1.1^{I311A}$	B834	vector for IPTG inducible expression of fHbp $V1.1^{I311A}$	[1]
pET28a_V2.24_A	B834	vector for IPTG inducible expression of fHbp $V2.24^{261R}$	This study
pET28a_V2.24_G	B834	vector for IPTG inducible expression of fHbp $V2.24^{261G}$	This study
pUC19	DH5α	Cloning vector	This study
pUC19*fHbp*$_{S-7}$T/$_{S13}$G	DH5α	Generating construct	This study
pUC19*fHbp*$_{S-7}$T/$_{S13}$A	DH5α	Generating construct	This study
pUC19 *fHbp*$_{S-7}$C/$_{S13}$G	DH5α	Generating construct	This study
pUC19 *fHbp*$_{S-7}$C/$_{S13}$A	DH5α	Generating construct	This study
pUC19*fba*$_{S897}$T/$_{S900}$C	DH5α	Generating construct	This study
pUC19*fba*$_{S897}$C/$_{S900}$C	DH5α	Generating construct	This study
pUC19*fba*$_{S897}$T/$_{S900}$T	DH5α	Generating construct	This study
pUC19*fba*$_{S897}$C/$_{S900}$T	DH5α	Generating construct	This study

Reference

1. Johnson S, Tan L, van der Veen S, Caesar J, Goicoechea De Jorge E, Harding RJ, et al. Design and evaluation of meningococcal vaccines through structure-based modification of host and pathogen molecules. PLoS Pathog. 2012;8(10):e1002981. Epub 2012/11/08. doi: 10.1371/journal.ppat.1002981. PMID: 23133374; PMCID: PMC3486911.
