# Supplementary material for: Genome-wide association studies reveal the role of polymorphisms affecting factor H binding protein expression in host invasion by Neisseria meningitidis
Source: PLoS Pathog. 2021 Oct 18;17(10):e1009992. doi: 10.1371/journal.ppat.1009992 (PMC8553145; doi:10.1371/journal.ppat.1009992)
Supplement: S3 Table — (PDF) [file ppat.1009992.s018.pdf]

S3 Table: Oligonucleotide Primers

Name	Sequence
pET28a-HIS-MBP-TEV-F	ACTTTAAGAAGGAGATATACCATGGGCCATCACCATCACCATC
pET28a-HIS-MBP-TEV-R	CGAGTGCGGCCGCAAGCTTGTCGACCCTGAAAATACAGATTTTCGCTACCCGGAGTCTGCGC
pET28a-GST-F	CTGGTGCCGCGCGGCAGCCATATGTCCCCTATACTAGGTTATTG
pET28a-GST-R	AGCTTTCTCCTTTTGGAGGATGGTCGC
pET28a-TEV-F	TCCTCCAAAAGGAGAAAGCTTGTTTAAGG
pET28a-TEV-R	GTCGACGGAGCTCGAATTCGGATCCTTAAGAACCAGGTTCTTC
pET21b_2.24_SNP_F	GCcatatgatggccgccgacatCGGCGCGGGGCTT
pET21b_2.24_SNP_A_R	GCctcgagctgtttgccggcGATGCCGATTTCGTGAACCTTTTCCCTTATCTTCACGGTTGC
pET21b_2.24_SNP_G_R	GCctcgagctgtttgccggcGATGCCGATTTCGTGAACCTTTTCCCCTATCTTCACGGTTGC
V2fHbp_pET28a_MBP_Tev_F	aaatctgtattttcagggtGTTGCCGCCGACATCGGCG
V2fHbp_ pET28a_MBP_Tev_R	agtggtggtggtggtggtgctcgagTTACTGTTTGCCGGCGATGCC
ML428	GATCCTCTAGAGTCGACCTGCAGGCATGCAAAAGTCATCAACGAATATGGC
ML429	AGAAACGAATCTGTATTTTAATTTGTCCGAGGGCGGTATGGCGCAAAAATG
ML430	TCGGACAAATTAAAATACAGATTCG
ML435	GCCGTGCCGTCGTGTCCTGGTACACGAAAAACAAGTTAAG
ML433	CAGGAAACAGCTATGACCATGATTACGCCAGGGCGATTTTGTTGCGGACG
ML434	CTTGTTTTTCGTGTACCAGGACACGACGGCACGGC

ML436	GTGAACCGAACTACCTTCTGCTGCCTTTTCCTGAC
ML437	GAAAAGGCAGCAGAAGGTAGTTCGGTTCACAGGTTTACTC
ML438	ATGACTAGGAGCAAACCTGTGAACCGAACTGC
ML439	AGTTCGGTTCACAGGTTTGCTCCTAGTCATACACAGAATAG
ML440	TAGGAGCAAACCTGTGAACCGAACTACCTTCTGCTGCCTTTTCCTG
ML441	AAGGTAGTTCGGTTCACAGGTTTGCTCCTAGTCATACACAGAATAG
ML405	TCCCGGCAACAATTAATAGAC
ML406	CCAGTCTATTAATTGTTGCCGG
ERS001	cgacgttgtaaaacgacggccagtgaattcCCGTGGTTAATTTCTCCCAC
ERS002	ggtatttgcgcggaTCAAAGTCGGACGGGTTTTC
ERS003	cgtccgactttgatCCGCGCAAATACCTGAGC
ERS004	tcgatgatggttgGGCGCAAAAATGTTACTGTTTG
ERS005	acatttttgcgccCAACCATCATCGATGAATTGTG
ERS006	cctagggcggtatCGATGCATGCCAACAGATAAAAC
ERS007	ttggcatgcatcgATACCGCCCTAGGACACGAC
ERS008	aagcttgcatgcctgcaggtcgactctagaGGGCAATGTCTGCCGCCC
ERS009	ATTTGCGCGGGTCGAAGTCGGACGGGTTTTCG
ERS010	CGTCCGACTTCGACCCGCGCAAATACCTGAGC
ERS011	ATTTGCGCGGATCGAAGTCGGACGGGTTTTCG
ERS012	TCGGTTCACAGGTTTACTCCTAGT
ER013	CAAAGTCAACATCGACACCGACC
GV1	TTCCGCCGCCTCCGCTGCTGCA
GV2	GCGGTCAGAATCAGGGCGGTGG
GV3	GGTTTACTCCTAGTCATACACAGA
