# Supplementary material for: Genome-wide association studies reveal the role of polymorphisms affecting factor H binding protein expression in host invasion by Neisseria meningitidis
Source: PLoS Pathog. 2021 Oct 18;17(10):e1009992. doi: 10.1371/journal.ppat.1009992 (PMC8553145; doi:10.1371/journal.ppat.1009992)
Supplement: S4 Table — (PDF) [file ppat.1009992.s019.pdf]

S4 Table: Sequences used for SHAPE analysis

Name	Sequence
fHbp$_{s-7}$T/	UUUUUGCUUC UUUGACCUGC CUCAUUGAUG CAAUAUGCAA
fHbp$_{s13}$G	AAAAAGAUAC CGCAACCAAA ACGUUUAUAU AUUAUCUAUU
	CUGUGUAUGA CUAGGAGUAA ACCUGUGAAC CGAACUGCCU
	UCUGCUGCCU UUUCCUGACC ACCGCCCUGA UUCUGACCGC
	CUGCAGCAGC GGAGGCGGCG GAA
fHbp$_{s-7}$T/	UUUUUGCUUC UUUGACCUGC CUCAUUGAUG CAAUAUGCAA
fHbp$_{s13}$A	AAAAAGAUAC CGCAACCAAA ACGUUUAUAU AUUAUCUAUU
	CUGUGUAUGA CUAGGAGUAA ACCUGUGAAC CGAACUACCU
	UCUGCUGCCU UUUCCUGACC ACCGCCCUGA UUCUGACCGC
	CUGCAGCAGC GGAGGCGGCG GAA
fHbp$_{s-7}$C/	UUUUUGCUUC UUUGACCUGC CUCAUUGAUG CAAUAUGCAA
fHbp$_{s13}$G	AAAAAGAUAC CGCAACCAAA ACGUUUAUAU AUUAUCUAUU
	CUGUGUAUGA CUAGGAGCAA ACCUGUGAAC CGAACUGCCU
	UCUGCUGCCU UUUCCUGACC ACCGCCCUGA UUCUGACCGC
	CUGCAGCAGC GGAGGCGGCG GAA
fHbp$_{s-7}$C/	UUUUUGCUUC UUUGACCUGC CUCAUUGAUG CAAUAUGCAA
fHbp$_{s13}$A	AAAAAGAUAC CGCAACCAAA ACGUUUAUAU AUUAUCUAUU
	CUGUGUAUGA CUAGGAGCAA ACCUGUGAAC CGAACUACCU
	UCUGCUGCCU UUUCCUGACC ACCGCCCUGA UUCUGACCGC
	CUGCAGCAGC GGAGGCGGCG GAA
