# Supplementary material for: Genome-wide association studies reveal the role of polymorphisms affecting factor H binding protein expression in host invasion by Neisseria meningitidis
Source: PLoS Pathog. 2021 Oct 18;17(10):e1009992. doi: 10.1371/journal.ppat.1009992 (PMC8553145; doi:10.1371/journal.ppat.1009992)
Supplement: S5 Table — (PDF) [file ppat.1009992.s020.pdf]

S5 Table: Comparison of the alleles tested by Spinsanti et al. [1] to the strains we used to test *fHbp*$_{s\text{-}7}$C/T and *fHbp*$_{s13}$G/A

Spinsanti et al. allele variants compared to our experimental strains, adapted from Spinsanti *et al.* [1]. Variant positions relate to the positions used in Figure 2D where +1 begins at the first position of the *fHbp* start codon, and -1 begins at the first position of the upstream intergenic region. The eleven alleles tested by *Spinsanti et al.* are listed, followed by the strains we used to test the SNPs *fHbp*$_{s\text{-}7}$C/T and *fHbp*$_{s13}$G/A. Where the *Spinsanti et al.* alleles differ to our experimental strain the variant is shown in bold, except for at the sites we tested experimentally. The two upstream variants that we tested are highlighted with boxes, the variants underlined, and where the *Spinsanti et al.* alleles contain the GWAS disease-associated variant, they are coloured in red. ATR describes an AT-rich insertion element present in Spinsanti *et al.* allele fIR2 [1].

Study	Strain / allele	Spinsanti *et al.* [1] *fba* Rhoindependent terminator strength	Spinsanti *et al.* [1] fHbp expression group	Spacer variants				-10 box	-45	-43	-38	-32	ATR	-21	RBS	GWAS *fHbp*$_{s-7}$C/T	+6	GWAS *fHbp*$_{s13}$G/A aRBS-1	aRBS-2
				-73	-72	-71	-68	(-57 to -52)							(-12 to -8)	-7		(+11 to +16)	(+21 to +26)
**Spinsanti *et al.* alleles tested** [1]<br><br>**Background strain MC58, CC32, fHbp variant 1.1**	**fIR1**	Weak (Read-through identified)	High	A	**G**	T	G	TACCGC	A	C	A	T		T	AGGAG	T	C	CTACCT	CTGCCT
	**fIR7**	Weak (Read-through identified)	High	**G**	**G**	T	G	TACC**AT**	A	**T**	A	T		**C**	AGGAG	T	**T**	CTGCCT	CTGCCT
	**fIR11**	Strong	Medium	**G**	**G**	T	**A**	TACC**AT**	**G**	**T**	**T**	T		**C**	AGGAG	T	**T**	CTGCCT	CTGCCT
	**fIR16**	Medium (Read-through identified)	Medium	A	A	T	G	TACCGC	A	C	A	T		T	AGGAG	T	C	CTGCCT	CTGCCT
	**fIR6**	Strong	Medium	A	**G**	T	G	TACC**AT**	A	**T**	A	**C**		**C**	AGGAG	C	C	CTGCCT	CTGC**TT**
	**fIR3**	Strong	Low	A	**G**	T	G	TACC**AT**	A	**T**	A	T		**C**	AGGAG	T	C	CTGCCT	CTGC**TT**
	**fIR13**	Strong	Low	A	**G**	T	G	TACC**AT**	A	**T**	A	T		**C**	AGGAG	C	C	CTGCCT	CTGC**TT**
	**fIR2**	Strong	Low	A	**G**	T	G	TACC**AT**	A	**T**	A	T	**ATR**	**C**	AGGAG	C	C	CTGCCT	CTGCCT
	**fIR4**	Strong	Low	A	**G**	T	G	TACC**AT**	A	**T**	A	T		**C**	AGGAG	C	C	CTGCCT	CTGCCT
	**fIR15**	Strong	Low	A	**G**	T	G	TACC**AT**	A	**T**	A	T		**C**	AGGAG	C	C	CTACCT	**T**TGCCT
	**fIR20**	Strong	Low	A	**G**	**C**	G	TACCGC	A	C	A	T		T	AGGAG	T	C	CTGCCT	CTGCCT
**Strains used in this study to test SNPs *fHbp*$_{s-7}$C/T and *fHbp*$_{s13}$G/A**<br><br>**Background strain 0011/93, CC41/44, fHbp variant 3**	**0011/93**	NA	NA	A	A	T	G	TACCGC	A	C	A	T		T	AGGAG	T	C	CTGCCT	CTGCCT
	**0011/93 *fHbp*$_{s-7}$T/$_{s13}$G**	NA	NA	A	A	T	G	TACCGC	A	C	A	T		T	AGGAG	T	C	CTGCCT	CTGCCT
	**0011/93 *fHbp*$_{s-7}$T/$_{s13}$A**	NA	NA	A	A	T	G	TACCGC	A	C	A	T		T	AGGAG	T	C	CTACCT	CTGCCT
	**0011/93 *fHbp*$_{s-7}$C/$_{s13}$G**	NA	NA	A	A	T	G	TACCGC	A	C	A	T		T	AGGAG	C	C	CTGCCT	CTGCCT
	**0011/93 *fHbp*$_{s-7}$C/$_{s13}$A**	NA	NA	A	A	T	G	TACCGC	A	C	A	T		T	AGGAG	C	C	CTACCT	CTGCCT

Reference

1. Spinsanti M, Brignoli T, Bodini M, Fontana LE, De Chiara M, Biolchi A, et al. Deconvolution of intergenic polymorphisms determining high expression of Factor H binding protein in meningococcus and their association with invasive disease. PLoS Pathog. 2021;17(3):e1009461. Epub 2021/03/27. doi: 10.1371/journal.ppat.1009461. PMID: 33770146; PMCID: PMC8026042.
