# Supplementary material for: Genome-wide association studies reveal the role of polymorphisms affecting factor H binding protein expression in host invasion by Neisseria meningitidis
Source: PLoS Pathog. 2021 Oct 18;17(10):e1009992. doi: 10.1371/journal.ppat.1009992 (PMC8553145; doi:10.1371/journal.ppat.1009992)
Supplement: Attachment — Submitted filename: Resubmission letter.pdf [file ppat.1009992.s023.pdf]

UNIVERSITY OF OXFORD

**Martin C. J. Maiden, BSc, MA, PhD, FRCPath, FRSB, FMedSci**
*Professor of Molecular Epidemiology*
Department of Zoology, Peter Medawar Building, South Parks Road, Oxford, OX1 3SY, United Kingdom

Phone: +44 (0) 1865-271284 (Direct)
Phone: +44 (0) 1865-281549 (Office, Mrs Andrea Kastner)
E-mail: Martin.Maiden@zoo.ox.ac.uk
Andrea.Kastner@zoo.ox.ac.uk

Friday, 27 August 2021

The Editorial Board, PLOS Pathogens

Dear Editors,

**RE: PLOS Pathogens PPATHOGENS-D-21-01153, resubmission**

Very many thanks for reviewing this manuscript and inviting a resubmission in response to the reviewers' comments and suggestions. We have considered the suggestions and the revised manuscript is attached, which we hope adequately addresses each of them. In short, we agreed with the reviewers that the manuscript required elaborating in parts, specifically in the discussion of the GWAS results and the comparison with the Spinsanti *et al.* paper. This has resulted in an expanded manuscript with three major changes: (i) the inclusion of additional material in the introduction and discussion; (ii) the incorporation of text from the supplementary materials into the main article; (iii) a thorough revision of the reference list, which is substantially expanded to place the discussion of the major GWAS results into an appropriate context. We have taken the opportunity to review the entire manuscript and have made a number of minor changes throughout to improve readability and context in the light of the other revisions made: these are all visible in the marked up manuscript version.

Specific changes in response to the reviewer's comments are:

**Major Issues:**

**Reviewer #2:** 'The experiments on fHbp are well documented, although it is to be regretted that the authors did not address other SNPs and Kmers. Considering the early online publication of Spinsanti et al, I would not suggest further experiments. However, and considering the very short discussion, I would really appreciate that the authors discuss other GWAS association, especially those presented in Fig1:
- fba is an important finding. This gene was found to be essential in the blood of mice. However, the authors were only interested in fHbp. Modification of fba sequence may alter its metabolic function.
- pilV is an interesting finding since it has been proposed as an adhesin.
- mafB was described as par of a toxin-antiToxin system.
- lptF is involved in LOS maturation
- some genes are involved in RNA an tRNA maturation (gidA, vacB)
- trkH may regulate K+ uptake, which may be related to resistance against the host.'

To meet these comments:

- the introduction has been expanded (specifically new paragraph 4, lines 105-112 in the unmarked file);
- S1 Table ‘Summary of significant Kmer associations’ has been moved into the main manuscript as Table 1, which has been enhanced by the inclusion of alternative annotations and appropriate references;
- material describing each of the GWAS hits has been moved and edited from the supplementary to the main ‘results’ text (lines 143-189 in the unmarked file);
- two paragraphs have been added to the discussion (new paragraphs 2 & 3, lines 286-315 in the unmarked manuscript file);
- additional references have been added.

**Minor Issues:**

**Reviewer 2:** ‘1. The Discussion is very short and does not really compare and contrast between the two studies. This needs to be discussed and they need to go into detail. 2. They mention two other polymorphisms in the Results and show others in Figure 1 but do not mention the others in the Results nor any of these in the Discussion. The strength of this whole genome study relative to the focused Spinsanti paper is the identification of these other loci. They each should be given some attention in the Results and the Discussion.’

With respect to point 1:

- in the introduction we have included additional discussion of the approach we have used, in comparison to the candidate gene approach, and followed this up in the discussion;
- the discussion now includes a more detailed treatment of our results and comparison with the Spinsanti study, largely generated by moving and editing material form the former supplementary material

Point 2 is addressed by our response to Reviewer 1, as these are essentially the same issues.

We feel that these changes have substantially improved the manuscript, and hope that it is now suitable for publication.

With many thanks once again for the opportunity to revise this work,

Yours faithfully,

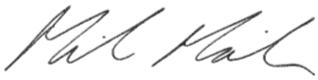

Martin C.J. Maiden
